# A Design Strategy for Energy-Efficient Rural Houses in Severe Cold Regions

**DOI:** 10.3390/ijerph17186481

**Published:** 2020-09-06

**Authors:** Wente Pan, Hongyuan Mei

**Affiliations:** 1 Heilongjiang Cold Region Architectural Science Key Laboratory, School of Architecture, Harbin Institute of Technology, Harbin 150001, China; meihongyuanadri@126.com; 2 Architectural Design and Research Institute of HIT, Harbin 150090, China

**Keywords:** low energy consumption, severe cold region, rural houses, design and construction, passive design, energy consumption analysis

## Abstract

In the past decade, Chinese urban areas have seen rapid development, and rural areas are becoming the next construction hotspot. The development of rural buildings in China has lagged behind urban development, and there is a lack of energy-efficient rural buildings. Rural houses in severe cold regions have the characteristics of large energy exchange, a long heating cycle, and low construction costs. Energy consumption is a crucial issue for rural houses in severe cold regions. How to balance the energy efficiency and building cost become a crucial problem. To solve this problem, we investigate the energy consumption of rural housing in cold regions, using Longquan Village in Heilongjiang Province, northeast China, as a case study. A low-energy design framework is established that considers the spatial layout, building type, enclosure system, and heating system. With the support of project funds, a demonstration house is constructed, and the energy savings performance of the building is investigated during the heating period. The results indicate that the energy savings rate of the demonstration house is 66%. The demonstration building enables local residents to learn construction methods for low-energy houses and promotes energy efficiency.

## 1. Introduction

With the improvement of urban construction in China and the shift of the construction focus to villages and towns, rural housing has become a hotspot in recent years. According to 2016 demographics, China’s rural population accounts for 46% of the total population [1]. Even if rapid urbanization continues, the proportion of the rural population is expected to be 38% in 2030 and 27% in 2050 [2], and the proportion of rural housing will remain high. China has highly variable climatic characteristics [3], and severe cold regions are areas where the average temperature of the coldest month is ≤−10 °C or the daily average temperature is ≤5 °C for ≥145 days [4]. These areas have a low sunshine duration in winter, large temperature differences throughout the year, and a long duration of low temperatures. Due to the extreme climate in severe cold regions, rural houses face adverse conditions such as low temperatures and cold winds. High energy input is needed to maintain a comfortable indoor environment, and a reduction in building energy consumption is an important goal in building design. Due to the stagnation of agricultural production in winter, residents in cold regions will engage in long-term indoors activities. However, due to the cold climate, outdoor activities are limited, and most rural residents remain indoors during this period. Therefore, it is vital to increase the energy efficiency of rural houses, reduce energy costs, and improve the indoor environment of rural houses to improve the quality of life of rural residents in severe cold regions.

### 1.1. Current Status of Rural Residential Energy Consumption in Severe Cold Regions

**Figure 1:**
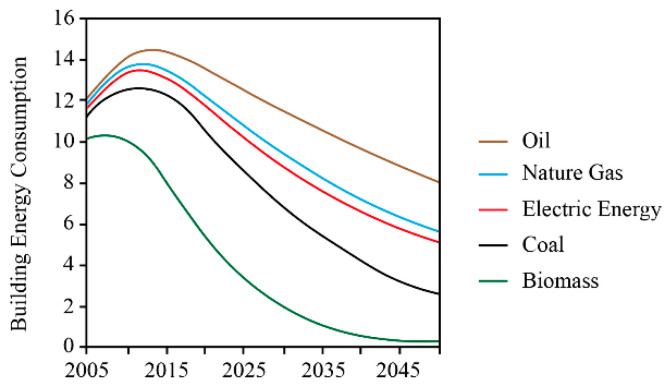
Energy type and consumption of rural residential buildings in cold regions.

High energy consumption and low energy efficiency are the primary problems associated with rural houses in cold regions. In 2005, 65% of building energy consumption came from rural areas [5]. Due to urbanization, the energy consumption of rural buildings accounted for 25% of total building energy consumption in 2017 [6]. Owing to poor design and technical constraints, the current energy-savings performance of rural houses in cold regions is low [7], and the construction of new rural houses in severe cold regions falls far short of the 65% energy-saving standard used in urban buildings. Rural houses use primarily traditional biomass and coal for heating [7], whereas new energy sources, such as natural gas and electrical energy, have not been widely used (Figure 1). The combustion efficiency of biomass and coal is low, resulting in high energy consumption and waste of energy [8]. Common energy sources for rural houses in cold regions include coal, traditional biomass, natural gas, new biomass, electrical energy, solar energy, and wind energy. Traditional biomass has low combustion efficiency and results in high air pollution; natural gas represents clean energy and is the future trend of rural heating. However, at present, natural gas has not been popularized in rural areas in northern China, and the current natural gas resources are insufficient. In addition, there are shortages, and a continuous and stable supply is not guaranteed. The new biomass fermentation technology requires a high temperature, which is limited in cold areas, and the fermentation efficiency is low. Electric membrane heating is a new option, but the cost of heating is 2–3 times that of coal, and there is a high risk of fire. Solar energy is widely used in mild climate regions, but there are still problems in cold regions. First, the construction cost is high, and the efficiency is low, which is not suitable for the promotion of solar energy. Second, in cold areas, solar equipment is covered by snow, which is not easy to remove. In addition, solar equipment is easily damaged by winter frost, and maintenance is also a problem. Wind energy is unstable and cannot be used continuously. As a result, although coal has low combustion efficiency and causes air pollution, it is still a suitable energy source that has advantages in terms of cost and stability.

**Figure 2:**
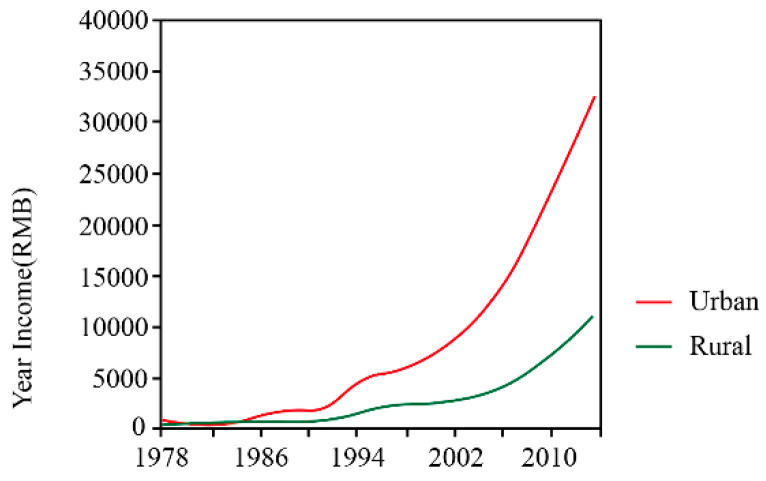
Urban and rural income.

Economic factors also affect the construction of rural housing in severely cold areas. Compared with urban areas, the rural economy is less developed. In 2014, the average annual income of the rural population in China was about 12,000 yuan, accounting for about one-third of the income of the urban population (Figure 2). However, China has extensive areas of cold regions, which are sparsely populated, and the economic development in these regions has lagged behind that of urban areas.

Indoor temperature fluctuations caused by the type of energy supply are another problem of rural houses in cold regions. Most residential buildings in northern China use intermittent heating, resulting in significant indoor temperature fluctuations in rural residential areas in cold regions. From 01:00 to 06:00, the indoor temperature is commonly below 10 °C [9], which is far from a comfortable indoor temperature. The indoor temperature fluctuations affect the health of the occupants and can cause physiological diseases in the elderly and children. The long-term indoor activities in winter exacerbate this effect.

### 1.2. Existing Energy-Saving Design Method for Rural Houses in Severe Cold Regions

Traditional rural houses in severe cold regions have experienced a long period of development, and high-efficiency passive technologies have been developed, such as the traditional Kang and the firewall [10]. Some of these traditional technologies are still used in current designs. However, traffic congestion, low economic development, and lack of information exchange in rural areas have resulted in the abandonment of traditional rural passive technology. As a result, these traditional technologies have not been well developed and implemented, and some traditional technologies are no longer suitable in current conditions. In recent years, with the promotion of rural projects in the country, rural areas have become construction hot spots, and new design and construction methods have been developed for rural houses in severe cold regions. Jin and Ling proposed an external wall structure of rural houses in severe cold regions that could adapt to the climate throughout its life cycle [11]. Zhu et al. proposed a multi-objective optimization system for rural houses in severe cold regions that focused on indoor comfort, the use of natural sunlight, and low energy consumption [6]. Liu et al. conducted research on the use of passive solar energy for rural residential areas in Qinghai, China [12]. Wang et al. investigated a thermal buffer space for houses in severely cold regions [13]. A window consisting of phase change material (PCM) was developed to improve the heat storage performance of houses [14]. These methods have achieved remarkable results and have promoted the development of rural housing in severe cold regions. However, there is a lack of research on energy-saving designs of rural houses, which complicates the integration of multiple energy-saving methods.

**Figure 3:**
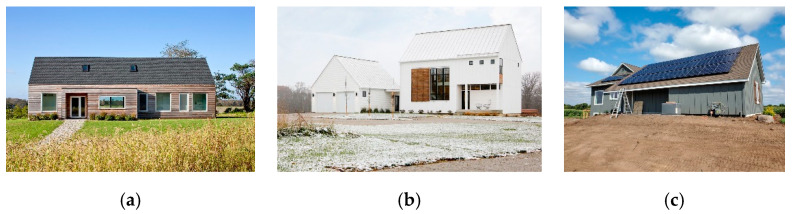
Examples of rural houses with low energy consumption in cold regions in the USA: (**a**) New England house [15], (**b**) Indianapolis house [16], (**c**) Afton house [17].

Due to global warming, many countries have focused on building energy consumption, and many types of low-energy housing have emerged (Figure 3). For example, the New England Rural Housing Project used passive design methods to achieve ultra-low indoor and outdoor heat transfer, with insulation values of R 40 in the wall, R 60 in the roof, and U 0.13 for the windows, winning the LEED Gold Award [15]. A rural house in Indianapolis was constructed by a couple and achieved low energy consumption [16]. Rural homes in Afton, MN, USA, were built with high-performance energy-saving materials to achieve extremely low building energy consumption [17]. These cases have significance for the design and construction of houses in severe cold regions in China, but several problems have to be addressed. First, these low-energy houses are mostly experimental projects with high construction costs and are not suitable for use in rural China. Second, it is necessary to use local materials and local construction methods since some foreign construction techniques cannot be implemented locally.

Therefore, rural houses in severe cold regions still face the problems of the lack of development of traditional energy-saving methods, the lack of systems to implement these methods locally, and the economic and regional constraints of using foreign energy-savings techniques. Therefore, in this study, we propose a design framework for rural houses that considers four aspects: the spatial layout, the building type, the enclosure system, and a heating system with low energy consumption, low cost, and easy construction in rural areas in cold regions.

## 2. Design Strategy for Rural Houses in Severe Cold Regions

The construction of rural houses in cold regions is constrained by various conditions, including a severe climate and environmental, economic, and technical constraints. Faced with many constraints, the objective of this study is to design a rural house that can be widely promoted in severe cold regions in China. A survey was conducted in Longquan Village, Wudalianchi area, and a questionnaire was developed focusing on basic information, functional requirements, the physical environment, and energy consumption (Appendix A Table A1).

### 2.1. Spatial Layout

**Figure 4:**
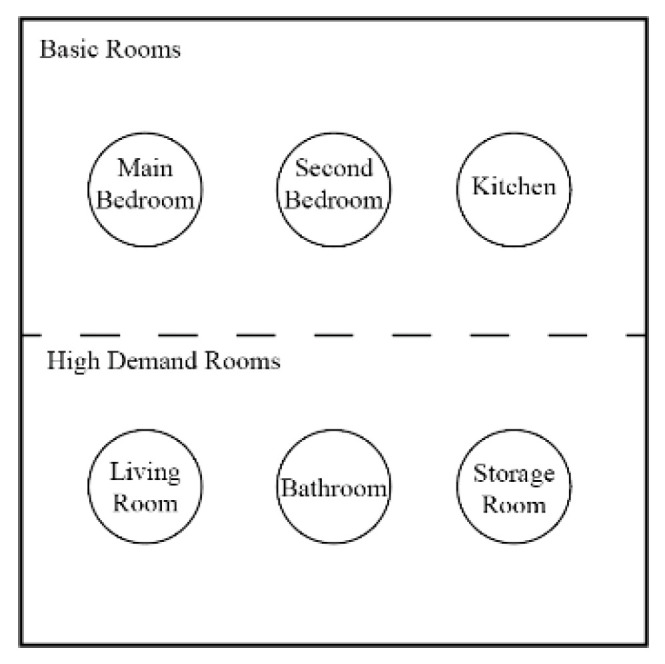
Primary functions.

**Figure 5:**
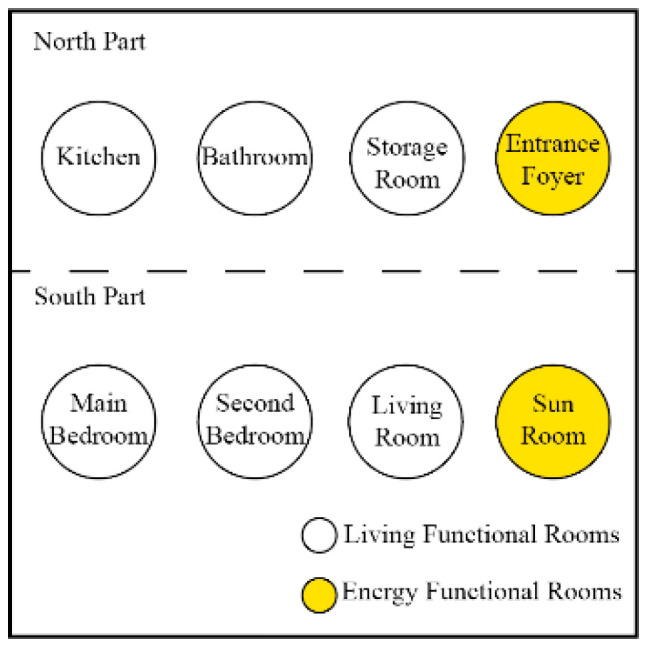
Demand functions.

The core function of rural housing is to provide a home for rural residents. An efficient spatial layout guarantees the functionality of the buildings and reduces energy consumption. The functional space of rural houses in severe cold regions consists of a primary space and a demand space. The primary space refers to the locations required for primary functions, and the demand space provides additional features that improve the lives of rural residents in the new era. The primary functional space is determined from the survey (Figure 4), and the demand space is designed based on the questionnaire (Figure 5).

**Figure 6:**
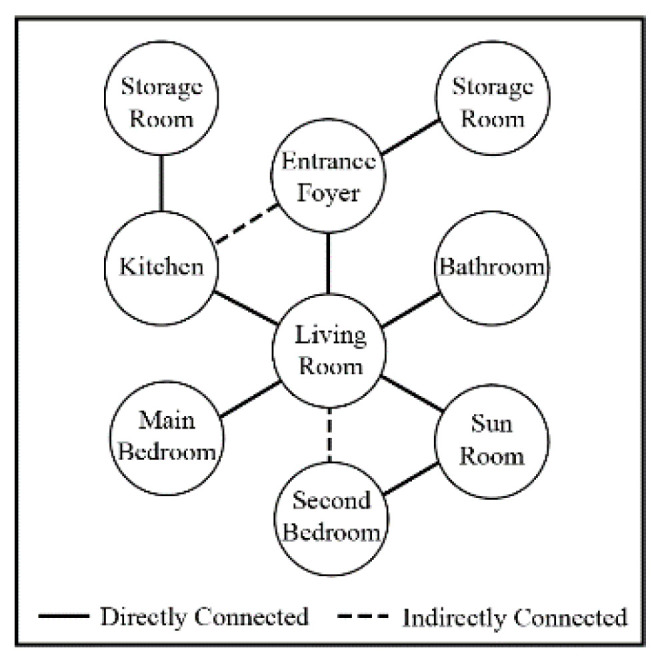
Organization of functions.

**Figure 7:**
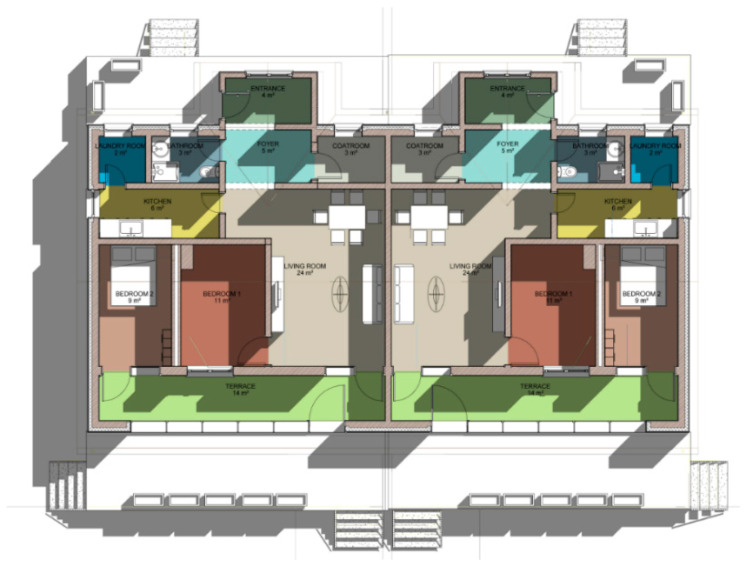
Layout of the test building.

Based on the primary functions and demand functions, the spatial layout of the building is designed. Rooms that are used extensively are located in the southern part of the building to receive more light, and the rooms that are used less are located in the northern part, which receives less sunlight. In addition, a foyer is located at the north entrance of the building as a heat buffer space to block the cold wind in the winter and reduce the heat exchange between the indoor and outdoor environments. As another heat buffer, a sunroom is located in the southern part of the building as a buffer and to improve the quality of the indoor environment. The functional organization diagram of the rural house in the cold region is shown in Figure 6, and the plan of the demonstration building is developed according to the diagram in Figure 7.

### 2.2. Building Type

The building type of rural houses in cold regions has a significant influence on energy savings, and the energy-saving performance of the building type can be determined by the shape coefficient. The shape coefficient C refers to the ratio of the external surface area F0 to the volume V0, i.e., C = F0/V0. The larger the C value, the worse the energy savings performance is, and vice versa.

**Figure 8:**
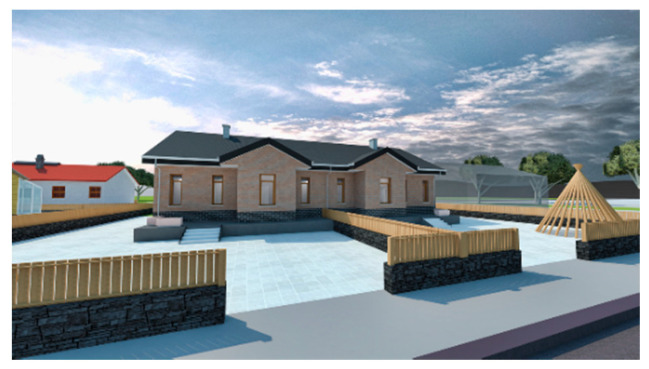
North façade of the demonstration building.

**Figure 9:**
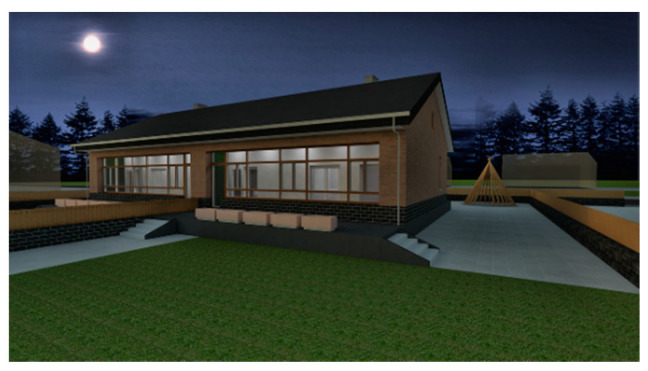
South façade of the demonstration building.

We consider the construction environment of Chinese houses in cold regions, the regulations for the construction of rural houses, as well as the promotion of these types of buildings in our design. Therefore, we designed a one-story house with 100 m^2^ per person in the household and two units side by side in the same building. The area of each single-family unit is 10 m × 10 m in length and width, and the two units are connected to form a 10 m × 20 m building. The interior floor height of the building is 3 m. Due to snow conditions, the roof is sloped with an angle of 26°, and the roof height is 5.3 m. The building is shown in Figure 8 and Figure 9. The surface area of the building is 412 m^2^, the volume is 860 m^3^, and the shape coefficient is 0.48.

### 2.3. Enclosure System

The building’s enclosure consists of the roofing, exterior walls, doors, and windows. The enclosure shields the indoor environment from the cold climate and has a significant influence on building energy consumption.

#### 2.3.1. Walls

Three types of external wall insulation are commonly used: internal insulation, middle insulation, and external insulation (Table 1). In rural houses in cold regions, the heat storage performance of the internal insulation method is poor, and condensation occurs between the insulation and the outer wall. As a result, the insulation performance is poor, and this type of wall insulation is not recommended. Even though the middle installation provides good insulation performance, the cost is relatively high. In this study, the external insulation type is used. High-hardness expanded polystyrene (EPS) board is used as the insulation material. The wall of the building consists of 20 cm thick concrete blocks, and the externally set 10 cm modified thermal transmittance of the EPS board is 0.051 W/(m^2^ K). This method has the advantages of low construction and maintenance costs.

#### 2.3.2. Windows

Windows are weak points in the building envelope system. The area of the windows is an important factor affecting the energy-saving performance of buildings. Four types of windows are commonly used in cold regions: energy-saving plastic windows, aluminum alloy windows, bridge-cut-off aluminum alloy windows, and aluminum-clad wood windows (Table 2). For rural houses in cold regions, energy-saving plastic steel windows are most suitable considering the energy-savings performance and the cost. In this study, three-layer double-cavity central control glass energy-saving plastic steel windows are used, and the window heat transfer coefficient is 2.2 W/(m^2^ K).

#### 2.3.3. Roof

**Figure 10:**
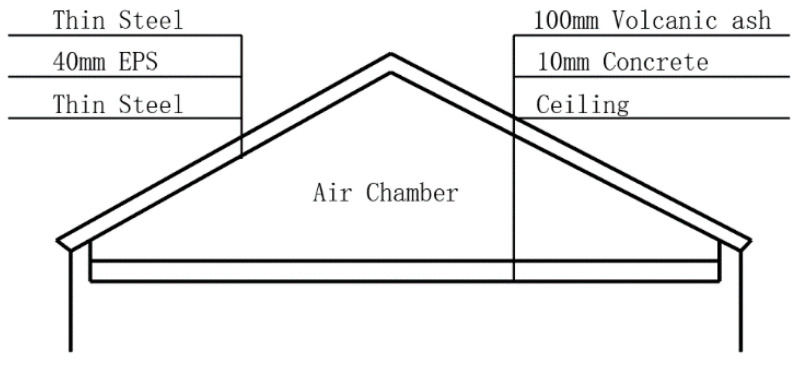
Details of the enclosed roof.

In this study, an “enclosed roof” (Figure 10) is used. The “enclosed roof” is a traditional roof type of rural houses in cold regions. It consists of a flat concrete roof as a support structure, a sloping roof to shed water and snow, and an air space in between. The air space reduces heat conduction and improves the energy savings performance. In this study, the traditional “enclosed roof” was improved. The sloped roof consisted of colored steel plates surrounding a 40 mm layer of EPS. The concrete roof was covered with a 10 cm layer of volcanic ash insulation material to improve the energy savings performance of the roof. Volcanic ash is a unique local material. EPS boards can be used in other areas.

### 2.4. Heating System

#### 2.4.1. Water Heating System Combined with the Traditional Kang and Firewall

**Figure 11:**
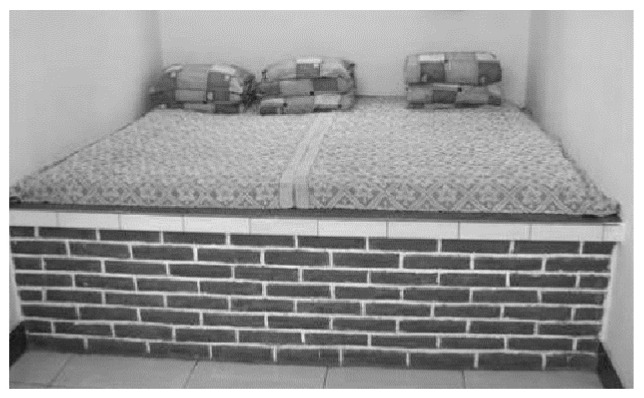
Traditional Kang in a rural house.

**Figure 12:**
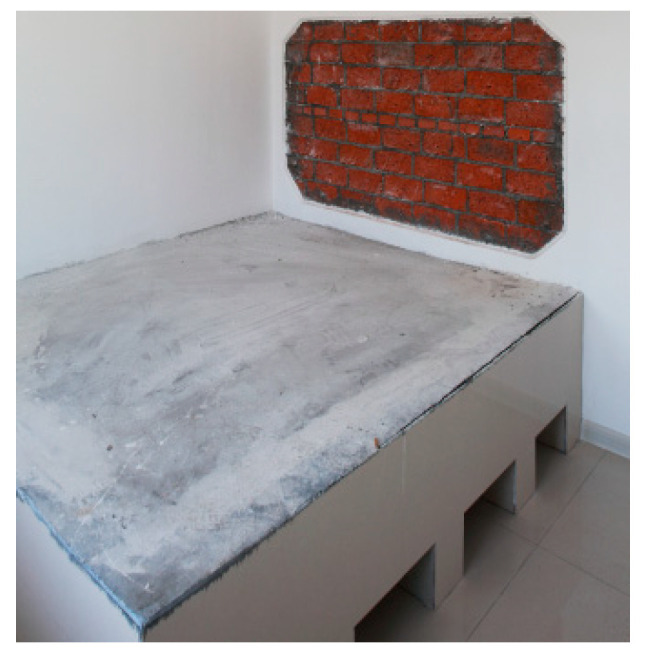
A hanging Kang in the demonstration building.

The Kang and firewall are traditional technologies used in rural houses in cold regions. The waste heat generated during cooking is channeled through a pipe to the Kang and firewall, where it is stored. The heat storage performance of the Kang is excellent, providing continuous heating. On the one hand, this technology maintains the indoor temperature at a low cost. On the other hand, the Kang is a necessary part of people’s lives in cold regions (Figure 11). However, Kang technology has the disadvantages of inconsistent heating. Therefore, a hanging kang is used in the demonstration house instead of a traditional Kang. The hanging Kang is raised off the floor, which increases the area of heat release (Figure 12). In addition, a water heating system is also incorporated in the system as a supplementary heating mode.

#### 2.4.2. Phase Change Heat Storage Device

**Figure 13:**
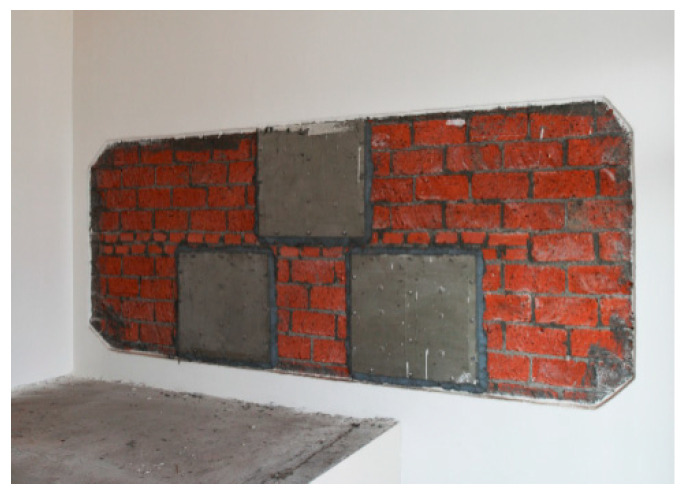
Phase change heat storage device.

**Figure 14:**
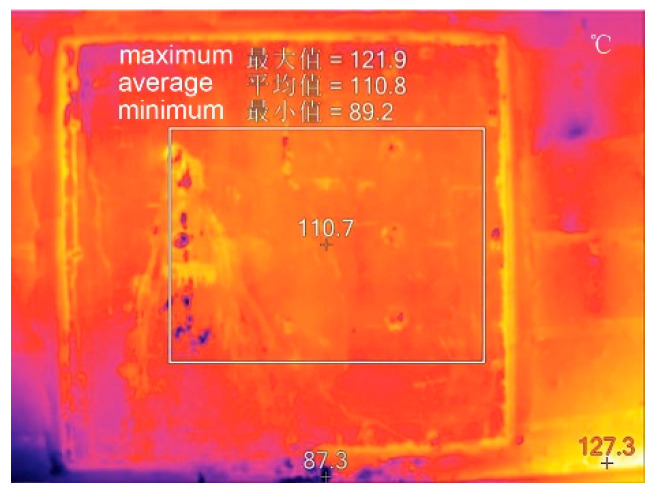
Test of the phase change heat storage device at 12 am.

A phase change heat storage device is used as a firewall in this study to improve the heat storage performance of the building. Phase change heat storage is a method for storing energy in materials that undergo changes in the state [18] to achieve continuous heat storage. Based on the research results of solid-liquid PCMs at the Harbin Institute of Technology [19], a phase change heat box was designed and placed on the firewall (Figure 13). The phase change box can store a large amount of heat during cooking and continues to release heat into the environment for 6–8 h, significantly extending the heating time of the traditional firewall (Figure 14).

## 3. Performance Comparison Test and Simulation Analysis of the Demonstration House

### 3.1. Demonstration House and Comparative House

**Figure 15:**
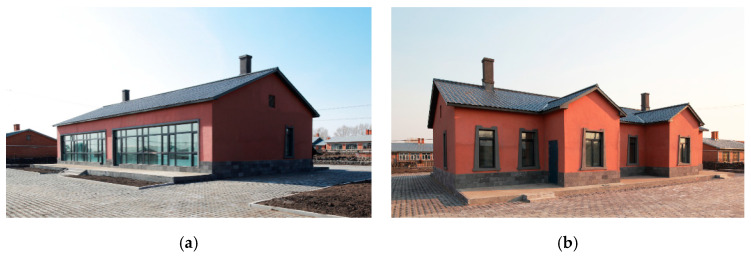
Outside views of the demonstration house. (**a**) South facade; (**b**) north facade.

**Figure 16:**
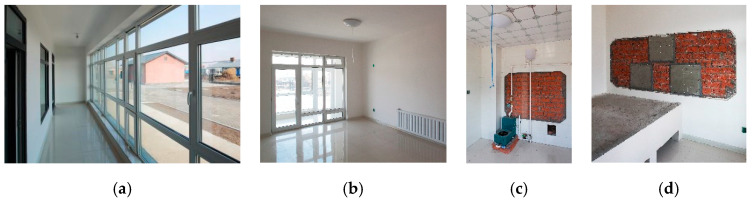
Inside views of the demonstration house. (**a**) Sunroom; (**b**) living room; (**c**).heating system; (**d**) phase change heat storage device in the firewall.

The demonstration house was constructed by local villagers with instruction from a professional construction team. The construction period lasted from 5 June 2015 to 7 September 2015, for a total of 93 days. The area of the completed building was 203.28 m^2^, and that of the single-family unit was 101.64 m^2^. The total cost was 246,800 yuan, the cost of the single-family unit was 123,400 yuan, and the cost per sq m was 1214 yuan. Figure 15 and Figure 16 show photos of the completed demonstration house.

A rural house in the same village that was constructed in 2008 was selected for the comparison with the demonstration house (Figure 15). Information on the two buildings is listed in Table 3. The comparative house is representative of houses in the Chinese northeastern rural area.

### 3.2. Test Methods

**Figure 17:**
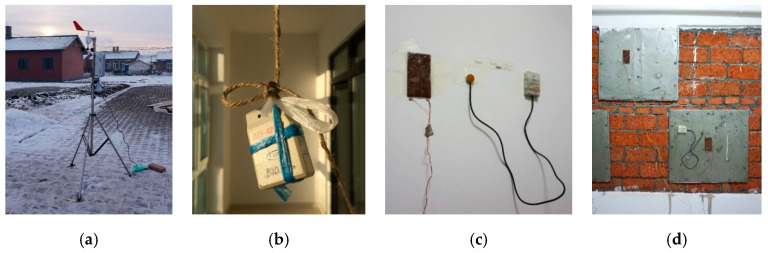
Test equipment layout. (**a**) Outdoor weather station on-site; (**b**) indoor thermometer in the main room; (**c**) heat flow meter on the interior wall; (**d**) thermometer and heat flow meter on the phase change heat storage device in the firewall.

The tests included measurements of the outdoor meteorological parameters, room temperature, thermal transmittance of the envelope structure, building energy consumption, and the heat storage capacity of the phase change heat storage device on the firewall. The tests were conducted according to the Chinese standard “JGJ/T132-2009 Residential Building Energy Conservation Inspection Standard” [20]. For the energy consumption test of heating in the winter, we considered the living habits and heating arrangements of the surveyed farmers. An auxiliary radiator and coal-fired water heater was used. The fire was extinguished at 21:00, and the fire was started at 06:00. The fuel for the Kang was wood, which was added at 06:30 and 17:30. The coal consumption and wood consumption were determined by weighing using electronic scales. The test equipment and layout are shown in Figure 17, and the details are listed in Table 4.

### 3.3. Test Results

#### 3.3.1. Room Temperature

**Figure 18:**
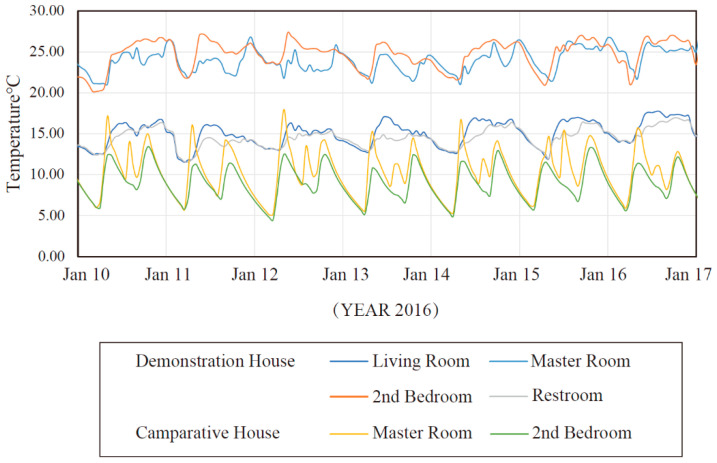
Temperature of the main room.

Figure 18 shows the air temperature fluctuations in the main rooms, including the living room, two bedrooms, and the bathroom in the demonstration house and the two bedrooms in the comparative house.

**Figure 19:**
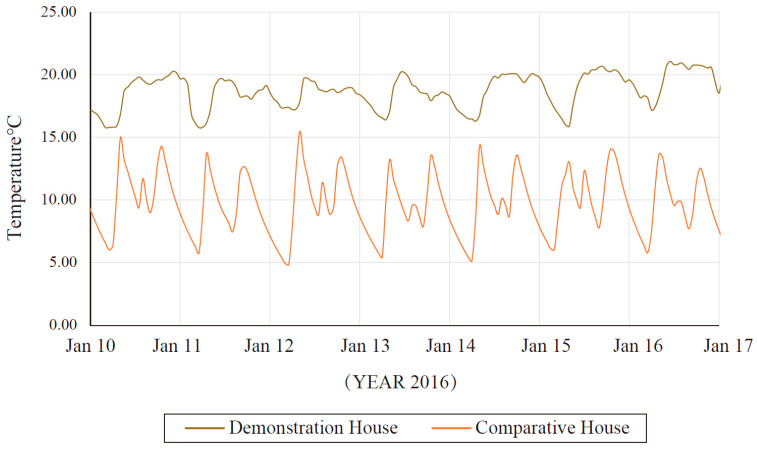
Temperature in the builidng.

Figure 19 shows that the general temperature in the demonstration building is higher than that of the comparative house, and the temperature fluctuations during the day and night are also significantly lower in the demonstration building. The specific reasons will be explained in conjunction with the follow-up experimental data. The thermal comfort of the demonstration house is significantly higher than that of the comparative house. For example, in the master room, the average temperature in the cycle is 23.76 °C, and the maximum temperature difference between day and night does not exceed 5 °C. The average temperature in the main bedroom of the comparison building is 10.37 °C, and the temperature difference between day and night exceeds 8 °C. The temperature was measured in each room in the demonstration building, and it was found that the temperature in the bedroom was higher than that of the other rooms. The temperature fluctuation was low because the bedroom has a Kang and firewall as an auxiliary heat source, providing high thermal comfort.

**Figure 20:**
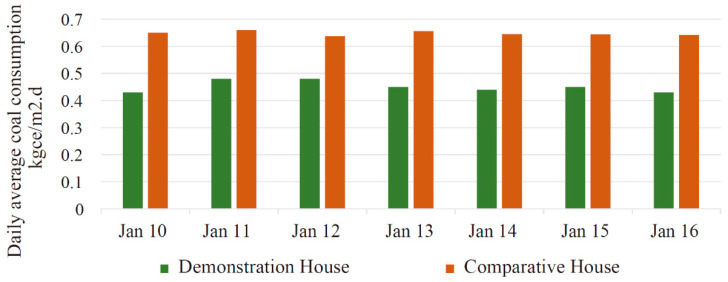
Comparison of the building energy consumption.

Figure 20 shows the average temperature in the two houses. The indoor temperature of the demonstration building is higher, and the temperature fluctuations are lower than in the comparative house. The average temperature of the demonstration building in the test period is 18.46 °C, and the maximum temperature difference between day and night does not exceed 5 °C. The temperature in the comparative house is 9.78 °C, and the temperature difference between day and night exceeds 7 °C. It is observed that the minimum average indoor temperature of the two houses occurs between 05:00 and0 7:00. Subsequently, the temperature of the heated room increases. Due to the influence of solar radiation, the maximum average indoor temperature of the two houses generally occurs in the morning.

#### 3.3.2. Heat Transfer Coefficient of the Building Envelope

The thermal transmittance of the envelope structure of the demonstration house and the comparative house is measured for the non-transparent structure of the main part of the house. A heat flow meter method is used. Thermal transmittance are listed in Table 5. The “standard limit” represents the limit of the heat transfer coefficient of the envelope structure of rural residential buildings in severe cold regions in the standard “GB/T 50824-2013 Building Energy Conservation Design Standard” [21].

Table 5 shows that the heat transfer coefficients of the outer walls and suspended ceilings of the envelope of the model building are smaller than those of the comparison building and less than the standard limits. In addition, the coefficients of the comparison building do not meet the standard requirements. The windows in the demonstration building are three-layer glass-plastic steel windows with a heat transfer coefficient of 2.2 W/m^2^ K, as provided by the manufacturer. In contrast, the two single glass-framed windows with aluminum alloy frames in the comparative house have thermal insulation values that are far less than the standard heat transfer coefficient limit. Therefore, the energy-saving performance of the demonstration building exceeds the requirements of the national standards developed in 2013. In contrast, the results for the comparative building show that the existing rural houses require additional insulation to meet the standards.

#### 3.3.3. Building Energy Consumption

The two buildings use the same type of coal and wood. The average calorific value of coal is 2.6 × 104 kJ/kg, which is 0.89 kgce/kg when converted into standard coal. The average calorific value of wood is 0.41 kgce/kg. The heating methods in the two buildings include the Kang and firewall supplemented by a gravity-circulation hot water heating system, which uses coal and wood. For comparison, both fuels were converted into standard coal. Since the two buildings have different building areas, the standard coal consumption per unit building area is used for the energy consumption analysis. Since the indoor temperature of the comparative house is lower than that of the demonstration house, the energy consumption of the comparative house is modified using Equation (1):

(1) $$m^{\prime }=\frac{{t^{\prime }}_{n}-t_{w}}{t_{n}-t_{w}}\times m,$$

where *m′* is the correction value of the standard coal consumption per unit area per day of the comparative building, kgce/m^2^·d; m is the measurement value of the standard coal consumption per unit area per day of the comparative building, kgce/m^2^·d; $t_{n}^{\prime }$ is the daily average indoor temperature of the demonstration building, °C; $t_{n}$ is the average daily indoor temperature of the comparative building, °C; and $t_{w}$ is the average daily outdoor temperature, °C.

The comparison of building energy consumption during the test period is shown in Figure 20. When the two buildings use the same type of hot water boiler and fuel, the coal consumption per unit area of the demonstration house is less than that of the comparative house, and the average coal savings rate is 43.1%. There are three reasons. First, the demonstration house has better insulation performance than the comparative house, and doors and windows are better sealed, resulting in less heat dissipation. Second, the demonstration building makes more efficient use of thermal energy, and the heat storage wall significantly reduces coal consumption. Third, the hanging Kang and the firewall with the phase change device increase the waste-heat utilization.

There are significant performance differences between the heating systems of the two buildings. The test period is short, and the differences in the temperature of the two buildings may affect the test results. Therefore, it is necessary to conduct more in-depth research into the energy efficiency of the building using simulations in subsequent studies.

### 3.4. Simulation of the Annual Heating Energy Consumption of Model Buildings and a Comparison with Measured Data

**Figure 21:**
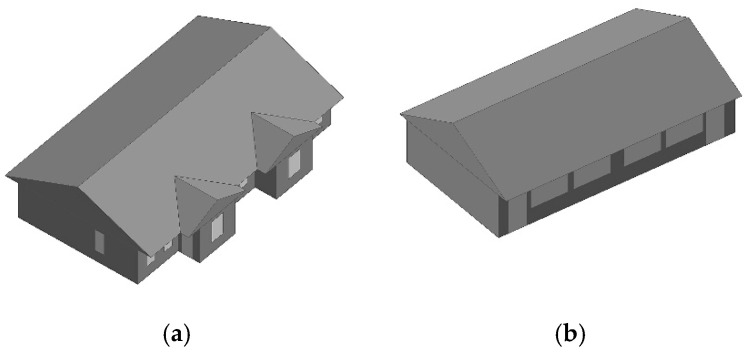
Building simulation model; (**a**) demonstration house; (**b**) comparative house.

A model was established based on the measurements (Figure 21), and a simulation was performed for different cold-region areas to investigate the performance of the demonstration building. Mohe, Keshan, and Shenyang were used as representative cities in regions with a severe cold climate. The simulation software is EnergyPlus, which was developed by the Lawrence Berkeley National Laboratory, Berkeley, CA, USA. The system uses a simulation method of integrated synchronous load/system/equipment. Table 6 lists the conductivity, specific heat capacity, and dry density of the main materials in the model, and Table 7 lists the thermal environmental control of the building in the heating period. In the simulation, the number of residents in the single-family unit in the two buildings was 3, and the number of air changes was 0.5 times/h. The domestic hot water sources are solar collectors and are not included in the building energy consumption. In the rural areas in the northeast, natural ventilation is used in the summer to reduce the room temperature, and air conditioners are rarely installed. Therefore, the summer cooling energy consumption is not included in the simulation. In both buildings, a hot water heating system is used in the winter, and the firewall is used to recover the waste heat of the flue gas. The fuel is converted to standard coal, and the boiler operating efficiency is 0.58. The design heating temperature of all rooms in the building is 18 °C. The reason for no control of the thermal environment between 21:00 and 06:00 in the bedroom of the model building is the use of the phase change device as heat storage, which ensures thermal comfort through heat storage.

## 4. Discussion

**Figure 22:**
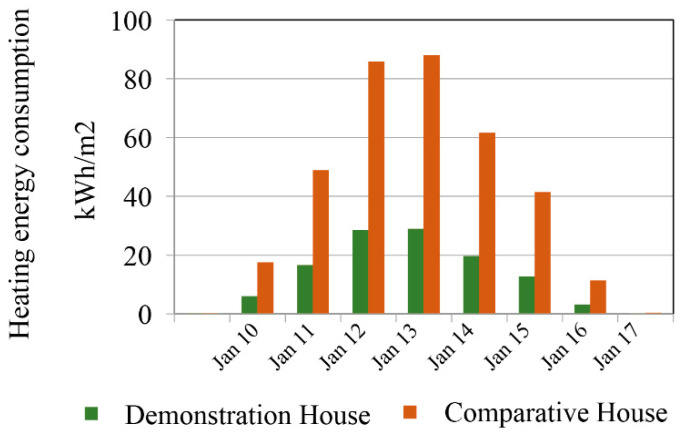
Monthly heating energy consumption per unit area of the two buildings in the Keshan area.

Figure 22 shows the energy consumption of the two buildings in the Keshan area. The heating energy consumption increases from October to January for both buildings, and the highest heating energy consumption in January is 87.9 kWh/m^2^ and 28.9 kWh/m^2^ for the comparative house and the demonstration house, respectively. Subsequently, the energy consumption decreases from January to April and is almost zero in May. However, the fluctuations of the monthly heating energy consumption are much lower for the demonstration building than the comparative building, indicating that the demonstration building meets the expected energy savings performance. The result shows a total energy saving rate of 67.3%. The results for Mohe and Shenyang are similar to those of Keshan. As shown in Table 8, as the latitude increases, the heating energy consumption of the buildings and the energy-savings rate increase, but the change in the energy-savings rate is very small, with a range of 66%–68% for the three regions. The results show that the demonstration house has significantly higher energy-savings than the demonstration house in severe cold regions.

**Figure 23:**
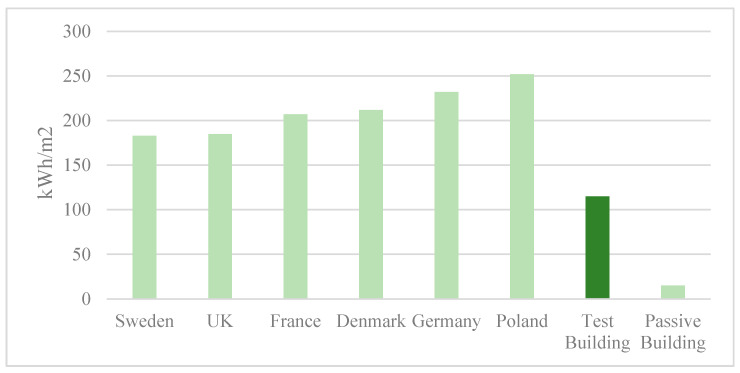
Heating energy consumption of the demonstration building and buildings in several countries.

The simulation results show that during the heating period from October to April, the total energy consumption of a single-family unit is 11,686.6 kWh, and the energy consumption per sq m is 115 kWh. According to the Odyssee database [22], the heating performance of the demonstration building exceeds the average performance of houses in regions with similar climates, such as Poland, Denmark, and Sweden [23] (Figure 23). Although there is a difference in the performance compared with the advanced energy-efficiency standard of passive buildings of 15 kWh/m^2^, the cost of 1214 RMB/m^2^ makes the demonstration building affordable for residents in rural areas in severe cold regions.

## 5. Conclusions

In this paper, we proposed a design framework for low-energy rural houses in severe cold regions, including the spatial layout, building type, enclosure system, and energy-efficient heating system. A demonstration house was constructed, and the energy efficiency of the building was investigated using field tests and simulations. The results showed that the green technologies incorporated in the demonstration building, such as high-performance envelopes, a heat buffer space, a phase change heat storage device as a firewall, and a hanging Kang, significantly improved the energy efficiency and the indoor thermal comfort. The demonstration house had an energy savings rate of 66% compared with local houses. The total heating energy consumption of the demonstration building was 115 kWh/m^2^, and the construction cost was 1214 yuan/m^2^.

Overall, a design strategy for rural house in severely cold region has been provided. The main foundling is for generating a framework to balance the energy efficiency and the construction cost for building a rural house in severely cold region. The use of the proposed design framework for rural houses can significantly reduce the energy consumption and carbon dioxide emissions, thereby reducing the economic burden on rural residents. In addition, the design framework has significance for demonstration projects and the promotion of energy efficiency.

## Figures and Tables

**Table 1:** Comparison of different types of wall insulation.

Type of Wall	Layers (from Internal to External)	d mm	Thermal Transmittance W/(m^2^ K)	Advantages	Disadvantages
Internal Insulation	Lime and cement plaster 10 mm EPS board 100 mm Brick 240 mm Lime and cement plaster 10 mm	320	0.72	Indoor temperature is easy to adjust	Poor heat storage, condensation can occur
Middle Insulation	Lime and cement plaster 10 mm Brick 120 mm Lime and cement plaster 10 mm EPS board 100 mm Lime and cement plaster 10 mm Brick 120 mm Lime and cement plaster 10 mm	340	0.86	Good protection of the thermal insulation layer; good heat storage performance	High cost
External Insulation	Lime and cement plaster 10 mm Brick 240 mm EPS board 100 mm Lime and cement plaster 10 mm	320	0.72	Good heat storage and low cost	Lack of protection for building insulation

**Table 2:** Comparison of different types of windows.

Type of Window	Advantages	Heat Transfer Coefficient W/(m^2^ K)	Cost Per sq m RMB
Energy-savings plastic steel window	Low cost and middle level of thermal insulation	2.0–3.4	180–300
Aluminum window	High strength and various opening methods	2.2–6.4	120–220
Broken bridge aluminum window	Good thermal insulation performance, lightweight and high strength	1.8–2.2	400–600
Aluminum-clad wood window	Thermal insulation, durable	0.8–1.5	1000–2500

**Table 3:** Information on the demonstration house and comparative house.

Item	Demonstration House	Comparative House
Photo	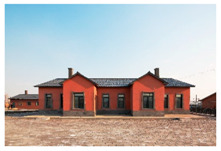	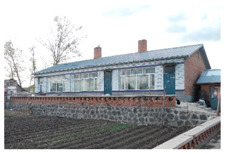
Year of completion	2015	2008
Floor area	101.48 m^2^	60 m^2^
Exterior wall structure	100 cm thick polystyrene board + 200 cm thick hollow concrete block wall	50 cm thick brick wall
Exterior windows	Three-layer glass-plastic steel windows	Aluminum window
Roof structure	Concrete board + 10 cm layer of volcanic ash + wooden roof frame + steel plate with 40 cm thick EPS boards	Wooden structure
Heating method	Water heating system + hanging Kang + phase change firewall	Water heating system + Kang + firewall
Heating fuel	Coal + wood	Coal + wood

**Table 4:** Test equipment.

Device Model/Name	Usage	Location
Small outdoor weather station	Outdoor weather parameters	Outdoors in a non-shaded area
Bes temperature probe	Room and wall temperature	Indoors at the center of the room
Heat flow meter	Heat flux	Inside the building on the exterior wall and the interior wall
T-scale electronic scale	Coal/biomass weight	
FLUKE infrared thermal imager FLK-TiS60	Temperature	

**Table 5:** Heat transfer coefficient of the building envelope.

Demonstration House		Comparative House		Standard Limit	
Wall	Roof	Wall	Roof	Wall	Roof
0.33	0.32	0.73	0.69	0.5	0.45

**Table 6:** Thermal conductivity, specific heat capacity, and dry density of the main materials in the simulation.

Material	Concrete	Polystyrene Board	Steel
Thermal conductivity (W/m.K)	1.740	0.030	58.200
Specific heat capacity (J/kg.K)	920.0000	5346.4000	480.0000
Dry density (kg/m^3^)	2500.00	25.00	7850.00

**Table 7:** Indoor thermal environment control of Demonstration House.

Time	Bedroom	Living Room, Kitchen, Rest Room	Foyer, Sunroom
6:00 to 21:00	18 °C	18 °C	No control
21:00 to 6:00	No control	No control	No control

**Table 8:** Heating energy consumption of the two buildings and energy savings rate in the three regions.

Project	Mohe	Keshan	Shenyang
Demonstration House	146.6	116.1	67.9
Comparative House	470.2	355.2	201.0
Energy savings rate (%)	68.8	67.3	66.2

## Appendix A

**Table A1:** Survey results.

Item	Sub-Item	House 1	House 2	House 3	House 4	House 5	House 6	House 7	House 8	House 9	House 10
Homeowner age		33	59	37	40	57	67	67	55	43	57
Family members		6	6	3	3	4	6	5	4	5	4
Residents		6	6	3	3	4	2	3	2	5	4
Annual income		50,000	25,000	15,000	20,000	10,000	10,000	7000	10,000	15,000	20,000
Generations		3	3	2	2	3	1	2	2	2	3
Energy cost		2600	2500	2000	3000	6000	2000	1500	3000	3000	1000
Structure type		Brick	Brick	Brick	Brick	Brick	Brick	Brick	Brick	Brick	Brick
Year		2008	1994	2008	1980	1996	1989	1996	1995	1992	1994
Cost		100 K	25 K	60 K	60 K	6 K	30 K	40 K	60 K	50 K	24 K
Floor		1	1	1	1	1	1	1	1	1	1
Indoor height		2.7 m	2.7 m	2.5 m	2.7 m	2.6 m	2.6 m	4 m	2.5 m	2.5	2.5 m
Roof height		1.8 m	1.5 m	4 m	4 m	1.25 m	4 m		4 m		
Total height		5 m	4 m	5 m	4 m	5 m	4 m	4.5 m	4 m	4.5 m	4.5 m
Building length		14 m	20 m	13 m	8.6 m	13.5 m	8.6 m	14 m	15.8 m		14 m
Building depth		7.6 m	8 m	8.3 m	7.5 m	8.5 m	7.8 m	8.25 m	8.5 m		8 m
Main bedroom	Area	18.4	24	9.43	30.4	8.37	12.4	19	20	15.48	26.8
	Length	4.8	4	4.1	7.6	2.7	3.1	5	5	4.3	6.7
	Depth	4	6	3.3	4	3.1	4	3.8	4	3.6	4
Second bedroom	Area	16.72		12.8	8.64	22	12.8	7.2	7.5	7.8	8.6
	Length	2.2		3.2	3.6	5.8	3.2	2.4	2.5	2.6	3.2
	Depth	7.6		4	2.4	3.1	4	3	3	6	2.7
Living room		√		√							
Dining room		√				√		√			
Restroom											
Storage				√		√					√
Balcony				√							
Fuel type	Summer	None	Wood	None	None	None	None	None	None	None	Wood
	Winter	Coal	Wood	Coal	Coal	Coal	Coal	Wood	Wood	Wood	Wood
Other fuel			CNG	CNG	CNG	CNG	Straw	CNG	CNG	CNG	CNG
